# Separator Wettability Enhanced by Electrolyte Additive to Boost the Electrochemical Performance of Lithium Metal Batteries

**DOI:** 10.1007/s40820-021-00731-2

**Published:** 2021-10-16

**Authors:** Ying Wang

**Affiliations:** Department of Chemistry, The Chinese University of Hong Kong, Shatin, N. T., Hong Kong SAR, People’s Republic of China

Lithium (Li) metal has been regarded as one of the most promising candidates to replace graphite anode due to its high theoretical specific capacity and the lowest electrochemical potential [1–3]. However, the immoderate growth of Li dendrite during Li plating/stripping causes serious safety problem and poor performance that severely impedes the practical application of lithium metal batteries (LMBs) [4–6]. Until now, there have been numerous kinds of strategies be proposed to inhibit Li dendrites growth and protect lithium metal anode such as high concentration electrolytes [7], construction of the solid electrolyte interface layer [8], structural design of anode materials [9], regulation of Li^+^ solvation [10], and solid-state electrolytes [11]. As an important part of battery structure, separator plays a vital role in the performance of battery [12]. The main function of separator is to divide the anode and cathode that prevents internal short circuit caused by direct contact between anode and cathode. So, the separator needs to be electrically insulated. At the same time, the separator also needs to ensure that the electrolyte is conductive between anode and cathode [13]. Therefore, it is necessary to render the separator fully wetted. Nevertheless, there are few researches on enhancing the wettability of the separator especially functional electrolyte additives.

**Fig. 1:**
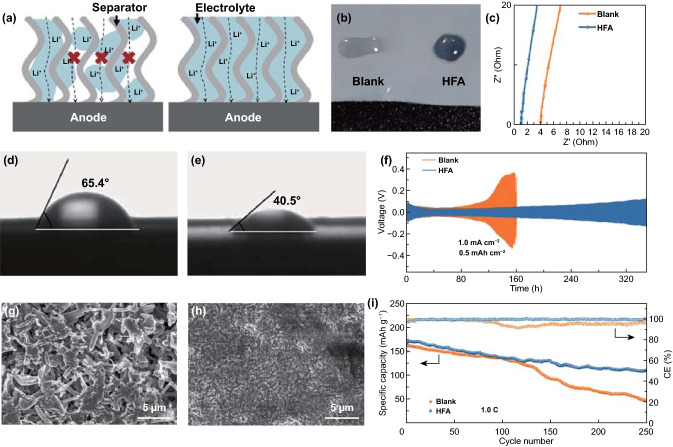
**a** Schematic illustration of the impacts of separator wettability toward electrolyte on Li^+^ transportation. **b** The photograph of different electrolytes dropped on the separator. **c** EIS result of SS||SS symmetric cells in different electrolytes. **d** The contact angles on separator for blank electrolyte. **e** The contact angles on separator for 1.0 wt% HFA-contained electrolyte. **f** The cycle performance of Li||Li symmetric cells in different electrolytes. **g** SEM image of Li anode after 50 cycles in blank electrolyte. **h** SEM images of Li anode after 50 cycles in 1.0 wt% HFA-contained electrolyte. **i** Cycling performance of Li||NCM622 cells in different electrolytes

Recently, Ma’s group conducted a detailed research and discussion on the study of separator wettability [14]. They employed heptafluorobutyric anhydride (HFA) as a multifunctional additive to modify the commercial electrolyte (1 M LiPF_6_ in EC/DMC 1:1). Benefited by the special chain structure, HFA can serve as the surfactant to promote the wetting of separator. Good wettability can make the separator easily to be wetted that facilitates the permeation of electrolyte. As shown in Fig. 1a, the schematic diagram visually describes the effect of different separator wettabilities toward electrolyte on Li^+^ transportation. The electrolyte must entirely fill the holes in the separator so that the channels for Li^+^ transferring can be built. The poor wettability of the electrolyte will cause some invalid channels in the separator that result in uneven Li^+^ flux for the whole Li metal surface. To assess the wettability, Ma’s group carried out the electrolyte uptake test and calculated the degree of electrolyte filling. After adding 1.0 wt% HFA, the electrolyte can wet the separator immediately, while the blank electrolyte forms into a droplet after dropping on the surface of the separator, as shown in Fig. 1b. In addition, the HFA-contained electrolyte uptake is 92.1%, much higher than 10.5% for blank electrolyte uptake. The degree of electrolyte filling increases from 11.1% in blank electrolyte to 97.3% in HFA-contained electrolyte, implying that the holes in separator have been sufficiently filled to build continuous pathways for Li^+^ flux. The poor wettability of separator also causes a higher resistance, resulting from the blocked paths for Li^+^ transportation (Fig. 1c). More intuitively, the introduction of HFA can reduce the surface tension of the electrolyte, reflecting in the smaller contact angle of electrolyte dropping on the separator, from 65.4° in blank electrolyte (Fig. 1d) reduce to 40.5° in HFA-contained electrolyte (Fig. 1e).

The sufficient Li^+^ transport channels can render uniform Li-ion flux, making the deposition of the lithium more uniform in the surface of anode. This will inhibit the growth of Li dendrites. As shown in Fig. 1f, the Li||Li symmetric cells assembled with HFA-contained electrolyte have excellent life performance that steadily cycles more than 320 h without severe polarization, while the cells with blank electrolyte rapidly fail only after 120 h. In addition, the surface of Li anode after 50 cycles in blank electrolyte is full of needle-like Li dendrites (Fig. 1g). In sharp contrast, there is no Li dendrite on the surface of anode cycling in HFA-contained electrolyte (Fig. 1h), implying a uniform deposition of lithium. Moreover, the addition of HFA also improves the cyclic stability performance of Li||NCM622 full cells, rendering higher capacity retention and Coulombic efficiency (CE), as shown in Fig. 1i.

In summary, this work from Ma’s group systematically and comprehensively explained the influence of separator wettability toward battery performance. The study on ion flux can also become a new research direction for LMBs to inhibit the growth of dendrite. In addition, they proposed the concept of electrolyte filling degree in separator, which could be a new index to study electrolytes in future.
